# Methyl 2-(2-{[(benz­yloxy)carbon­yl]amino}­propan-2-yl)-5-hy­droxy-6-meth­oxy­pyrimidine-4-carboxyl­ate

**DOI:** 10.1107/S160053681101628X

**Published:** 2011-05-07

**Authors:** Zhenhua Shang, Shan Qi, Xiao Tao, Guangbo Zhang

**Affiliations:** a College of Chemical and Pharmaceutical Engineering, Hebei University of Science and Technology, Shijiazhuang 050018, People’s Republic of China

## Abstract

In the title compound, C_18_H_21_N_3_O_6_, the dihedral angle between the two aromatic rings is 61.1 (1)°. The crystal structure is stabilized by inter­molecular O—H⋯O hydrogen bonds. An intra­molecular O—H⋯O hydrogen bonds is also present.

## Related literature

The title compound was obtained in an attempt to synthesise an inter­mediate for the anti­retroviral drug raltegravir [sys­tem­atic name *N*-(2-(4-(4-fluoro­benzyl­carbamo­yl)-5-hy­droxy-1-methyl-6-oxo-1,6-dihydro­pyrimidin-2-yl)propan-2-yl)-5-methyl-1,3,4-oxadiazole-2-carboxamide], see: Belyk *et al.* (2006) ▶. For background to raltegravir, see: Steigbigel *et al.* (2008 ▶). For related structures, see: Shang & Shang (2007 ▶); Fun *et al.* (2009 ▶).
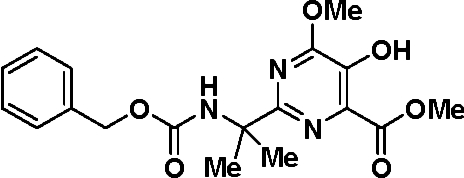


## Experimental

### 

#### Crystal data

C_18_H_21_N_3_O_6_
*M* *_r_* = 375.38
Monoclinic, 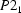

*a* = 8.5313 (17) Å
*b* = 6.5413 (13) Å
*c* = 16.167 (3) Å
β = 97.37 (3)°
*V* = 894.7 (3) Å^3^
*Z* = 2
Mo *K*α radiation
μ = 0.11 mm^−1^
*T* = 113 K
0.20 × 0.16 × 0.12 mm

#### Data collection

Bruker SMART CCD area-detector diffractometer
Absorption correction: multi-scan (*SADABS*; Bruker, 1997 ▶) *T*_min_ = 0.979, *T*_max_ = 0.987
7509 measured reflections
2281 independent reflections
1854 reflections with *I* > 2σ(*I*)
*R*_int_ = 0.044

#### Refinement

*R*[*F* ^2^ > 2σ(*F* ^2^)] = 0.039
*wR*(*F* ^2^) = 0.088
*S* = 1.01
2281 reflections
256 parameters
1 restraint
H atoms treated by a mixture of independent and constrained refinement
Δρ_max_ = 0.25 e Å^−3^
Δρ_min_ = −0.21 e Å^−3^

### 

Data collection: *SMART* (Bruker, 1997 ▶); cell refinement: *SAINT* (Bruker, 1997 ▶); data reduction: *SAINT*; program(s) used to solve structure: *SHELXS97* (Sheldrick, 2008 ▶); program(s) used to refine structure: *SHELXL97* (Sheldrick, 2008 ▶); molecular graphics: *SHELXTL* (Sheldrick, 2008 ▶); software used to prepare material for publication: *SHELXTL*.

## Supplementary Material

Crystal structure: contains datablocks global, I. DOI: 10.1107/S160053681101628X/hg5023sup1.cif

Structure factors: contains datablocks I. DOI: 10.1107/S160053681101628X/hg5023Isup2.hkl

Supplementary material file. DOI: 10.1107/S160053681101628X/hg5023Isup3.cml

Additional supplementary materials:  crystallographic information; 3D view; checkCIF report

## Figures and Tables

**Table 1:** Hydrogen-bond geometry (Å, °)

*D*—H⋯*A*	*D*—H	H⋯*A*	*D*⋯*A*	*D*—H⋯*A*
O2—H2⋯O5^i^	0.87 (3)	2.27 (3)	2.889 (2)	128 (2)
O2—H2⋯O3	0.87 (3)	1.95 (3)	2.652 (2)	136 (3)

Symmetry code: (i) 
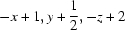
.

**Fig. 1.**
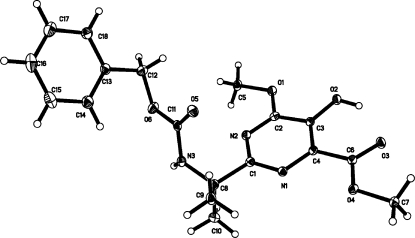
The molecular structure of the title compound, drawn with 30% probability ellipsoids. Hydrogen atoms are shown as spheres of arbitrary radius.

## supplementary crystallographic information

### Comment

Raltegravir (MK-0518, brand name Isentress) is an antiretroviral drug produced by Merck & Co, used to treat HIV infection (Steigbigel *et al.*, 2008). It received FDA approval in October 2007, the first of a new class of HIV drugs, the integrase inhibitors, to receive such approval. When methyl 2-(2-(benzyloxycarbonyl)propan-2-yl) -5-hydroxy-6-oxo-1,6-dihydropyrimidine-4-carboxylate was reacted with dimethyl sulfate catalyzed by magnesium methoxide in dimethyl sulfoxide (Belyk *et al.*, 2006), as we designed, in order to synthesize methyl 2-(2-(benzyloxycarbonyl)propan-2-yl) -5-hydroxy-1-methyl-6-oxo-1,6-dihydropyrimidine-4-carboxylate as the key intermediate of Raltegravir, two products appeared on thin layer chromatography. These products were separated through flash chromatography and the structures were conformed by nuclear magnetic resonance  and  X-ray analysis. The result showed that the title compound was the byproduct of the reaction. The pyrimidinone ring is planar, as it is in a related compound (Fun *et al.*, 2009). This is in contrast with another related compound (Shang *et al.*, 2007), where the heterocyclic ring is twisted. In the title compound the dihedral angle between the two aromatic rings is 118.9 (1)°. The crystal structure is stabilized through intermolecular O—H···O hydrogen bonds; intramolecular O—H···O hydrogen bonds are also present.

### Experimental

To a slurry of methyl 2-(2-(benzyloxycarbonyl)propan-2-yl) -5-hydroxy-6-oxo-1,6-dihydropyrimidine-4-carboxylate (1.5 g) and magnesium methoxide (2.1 g) in dimethyl sulfoxide(15 ml) at 70 °C, dimethyl sulfate (3.1 g) was added dropwise. After addition, the mixture was heated at the same temperature for 8 h. To the reaction mixture was then added 40 ml 2 N HCl and then 100 ml water. Solid appeared when the mixture was stirred in an ice-water bath. The products were filtered and separated by flash chromatography. 50 mg of the title compound was dissolved in 30 ml methanol and the solution was kept at room temperature for 10 d; natural evaporation gave colorless single crystals of the title compound suitable for X-ray analysis.

### Refinement

All H atoms attached to C atoms were fixed geometrically and treated as riding with C—H = 0.95 Å(aromatic), 0.98 Å(methyl group) or 0.99 Å(methylene group). *U*_iso_(H) = xUeq(C). where *x* = 1.5 for methyl H and 1.2 for all other carbon-bound H atoms. The positional parameters of the oxygen-bound H atoms and nitrogen-bound H atoms were refined freely(O-H=0.87 (3)Å, N-H=0.92 (3)Å).

### Crystal data

**Table:**

C_18_H_21_N_3_O_6_	*F*(000) = 396
*M**_r_* = 375.38	*D*_x_ = 1.393 Mg m^−^^3^
Monoclinic, *P*2_1_	Mo *K*α radiation, λ = 0.71073 Å
Hall symbol: P 2yb	Cell parameters from 3543 reflections
*a* = 8.5313 (17) Å	θ = 2.3–27.5°
*b* = 6.5413 (13) Å	µ = 0.11 mm^−^^1^
*c* = 16.167 (3) Å	*T* = 113 K
β = 97.37 (3)°	Plate, colorless
*V* = 894.7 (3) Å^3^	0.20 × 0.16 × 0.12 mm
*Z* = 2	

### Data collection

**Table:**

Bruker SMART CCD area-detector diffractometer	2281 independent reflections
Radiation source: rotating anode	1854 reflections with *I* > 2σ(*I*)
confocal	*R*_int_ = 0.044
Detector resolution: 7.31 pixels mm^-1^	θ_max_ = 27.9°, θ_min_ = 2.5°
ω and φ scans	*h* = −10→11
Absorption correction: multi-scan (*SADABS*; Bruker, 1997)	*k* = −7→8
*T*_min_ = 0.979, *T*_max_ = 0.987	*l* = −21→20
7509 measured reflections	

### Refinement

**Table:**

Refinement on *F*^2^	Primary atom site location: structure-invariant direct methods
Least-squares matrix: full	Secondary atom site location: difference Fourier map
*R*[*F*^2^ > 2σ(*F*^2^)] = 0.039	Hydrogen site location: inferred from neighbouring sites
*wR*(*F*^2^) = 0.088	H atoms treated by a mixture of independent and constrained refinement
*S* = 1.01	*w* = 1/[σ^2^(*F*_o_^2^) + (0.0491*P*)^2^] where *P* = (*F*_o_^2^ + 2*F*_c_^2^)/3
2281 reflections	(Δ/σ)_max_ = 0.001
256 parameters	Δρ_max_ = 0.25 e Å^−^^3^
1 restraint	Δρ_min_ = −0.21 e Å^−^^3^

### Special details

**Table:**

Experimental. 1*H*-NMR (500 MHz, CDCl3) 1.72(s, 6H), 3.66(s, 3H), 3.97(s, 3H), 5.03(s, 2H), 5.28 (s, 1H), 7.02–7.32(m, 5H, J=75 Hz), 10.39(s, 1H).
Geometry. All e.s.d.'s (except the e.s.d. in the dihedral angle between two l.s. planes) are estimated using the full covariance matrix. The cell e.s.d.'s are taken into account individually in the estimation of e.s.d.'s in distances, angles and torsion angles; correlations between e.s.d.'s in cell parameters are only used when they are defined by crystal symmetry. An approximate (isotropic) treatment of cell e.s.d.'s is used for estimating e.s.d.'s involving l.s. planes.
Refinement. Refinement of *F*^2^ against ALL reflections. The weighted *R*-factor *wR* and goodness of fit *S* are based on *F*^2^, conventional *R*-factors *R* are based on *F*, with *F* set to zero for negative *F*^2^. The threshold expression of *F*^2^ > σ(*F*^2^) is used only for calculating *R*-factors(gt) *etc*. and is not relevant to the choice of reflections for refinement. *R*-factors based on *F*^2^ are statistically about twice as large as those based on *F*, and *R*- factors based on ALL data will be even larger.

### Fractional atomic coordinates and isotropic or equivalent isotropic displacement parameters (Å^2^)

**Table:**

	*x*	*y*	*z*	*U*_iso_*/*U*_eq_	
N1	0.7129 (2)	0.6028 (3)	0.89047 (10)	0.0184 (4)	
N2	0.4395 (2)	0.6250 (3)	0.83861 (10)	0.0192 (4)	
N3	0.4877 (2)	0.4973 (3)	0.68708 (11)	0.0210 (4)	
H3	0.427 (3)	0.599 (5)	0.6595 (17)	0.043 (8)*	
O1	0.25348 (16)	0.6638 (3)	0.92818 (9)	0.0220 (4)	
O2	0.46916 (18)	0.6583 (3)	1.06058 (9)	0.0219 (4)	
H2	0.547 (3)	0.670 (6)	1.1011 (18)	0.050 (9)*	
O3	0.77719 (17)	0.6582 (3)	1.11167 (8)	0.0244 (4)	
O4	0.94697 (16)	0.6036 (3)	1.01878 (9)	0.0231 (4)	
O5	0.4667 (2)	0.2009 (2)	0.76039 (9)	0.0259 (4)	
O6	0.26425 (19)	0.3210 (3)	0.66809 (10)	0.0287 (4)	
C1	0.5941 (2)	0.6005 (3)	0.82953 (12)	0.0185 (5)	
C2	0.4043 (2)	0.6444 (3)	0.91420 (12)	0.0183 (4)	
C3	0.5207 (2)	0.6435 (3)	0.98553 (12)	0.0174 (4)	
C4	0.6744 (2)	0.6263 (3)	0.96913 (12)	0.0174 (4)	
C5	0.1359 (2)	0.6528 (4)	0.85502 (12)	0.0240 (5)	
H5A	0.1437	0.7746	0.8205	0.036*	
H5B	0.0302	0.6460	0.8725	0.036*	
H5C	0.1544	0.5304	0.8227	0.036*	
C6	0.8042 (2)	0.6304 (4)	1.04017 (12)	0.0193 (4)	
C7	1.0779 (3)	0.6187 (4)	1.08615 (13)	0.0258 (5)	
H7A	1.0933	0.7620	1.1029	0.039*	
H7B	1.1745	0.5663	1.0669	0.039*	
H7C	1.0537	0.5379	1.1340	0.039*	
C8	0.6296 (3)	0.5754 (3)	0.73967 (13)	0.0197 (5)	
C9	0.7695 (3)	0.4322 (4)	0.73342 (14)	0.0261 (6)	
H9A	0.7435	0.2947	0.7515	0.039*	
H9B	0.8627	0.4831	0.7693	0.039*	
H9C	0.7920	0.4271	0.6755	0.039*	
C10	0.6641 (3)	0.7866 (4)	0.70588 (15)	0.0285 (6)	
H10A	0.6796	0.7750	0.6470	0.043*	
H10B	0.7600	0.8422	0.7378	0.043*	
H10C	0.5749	0.8780	0.7111	0.043*	
C11	0.4121 (3)	0.3289 (4)	0.71076 (12)	0.0211 (5)	
C12	0.1749 (3)	0.1388 (4)	0.67933 (14)	0.0313 (6)	
H12A	0.0616	0.1746	0.6762	0.038*	
H12B	0.2092	0.0820	0.7355	0.038*	
C13	0.1953 (3)	−0.0221 (4)	0.61451 (14)	0.0248 (5)	
C14	0.3061 (3)	−0.0041 (5)	0.55949 (14)	0.0322 (6)	
H14	0.3742	0.1112	0.5625	0.039*	
C15	0.3181 (3)	−0.1545 (5)	0.49977 (15)	0.0399 (7)	
H15	0.3942	−0.1411	0.4621	0.048*	
C16	0.2197 (4)	−0.3238 (5)	0.49494 (16)	0.0427 (7)	
H16	0.2290	−0.4268	0.4544	0.051*	
C17	0.1087 (3)	−0.3423 (5)	0.54905 (15)	0.0379 (7)	
H17	0.0401	−0.4572	0.5455	0.046*	
C18	0.0970 (3)	−0.1928 (4)	0.60889 (14)	0.0293 (6)	
H18	0.0209	−0.2072	0.6465	0.035*	

### Atomic displacement parameters (Å^2^)

**Table:**

	*U*^11^	*U*^22^	*U*^33^	*U*^12^	*U*^13^	*U*^23^
N1	0.0175 (9)	0.0203 (10)	0.0177 (8)	0.0002 (8)	0.0028 (7)	−0.0018 (7)
N2	0.0165 (9)	0.0218 (9)	0.0190 (8)	0.0010 (8)	0.0020 (7)	−0.0023 (8)
N3	0.0206 (10)	0.0237 (10)	0.0178 (9)	0.0015 (8)	−0.0011 (7)	0.0003 (8)
O1	0.0129 (8)	0.0321 (9)	0.0211 (7)	0.0017 (7)	0.0021 (6)	−0.0023 (7)
O2	0.0196 (8)	0.0282 (9)	0.0176 (7)	0.0007 (7)	0.0020 (6)	−0.0022 (7)
O3	0.0215 (8)	0.0348 (10)	0.0170 (7)	0.0017 (8)	0.0035 (6)	−0.0023 (7)
O4	0.0126 (8)	0.0357 (10)	0.0209 (7)	−0.0004 (7)	0.0014 (6)	−0.0023 (7)
O5	0.0303 (10)	0.0250 (9)	0.0225 (8)	0.0032 (7)	0.0037 (7)	0.0021 (7)
O6	0.0249 (9)	0.0295 (9)	0.0295 (8)	−0.0040 (8)	−0.0044 (7)	−0.0018 (7)
C1	0.0180 (11)	0.0182 (11)	0.0193 (10)	0.0003 (9)	0.0024 (8)	−0.0012 (8)
C2	0.0161 (11)	0.0153 (11)	0.0236 (10)	0.0004 (9)	0.0032 (8)	−0.0011 (9)
C3	0.0208 (11)	0.0141 (10)	0.0174 (9)	−0.0015 (9)	0.0026 (8)	−0.0019 (8)
C4	0.0174 (11)	0.0164 (10)	0.0182 (9)	−0.0007 (9)	0.0017 (8)	−0.0012 (8)
C5	0.0176 (11)	0.0316 (13)	0.0214 (11)	0.0008 (10)	−0.0028 (8)	−0.0020 (10)
C6	0.0200 (11)	0.0191 (11)	0.0184 (10)	−0.0026 (10)	0.0009 (8)	−0.0002 (9)
C7	0.0190 (12)	0.0376 (14)	0.0196 (10)	−0.0044 (11)	−0.0016 (9)	0.0005 (10)
C8	0.0168 (11)	0.0253 (12)	0.0169 (10)	0.0001 (9)	0.0010 (8)	−0.0015 (8)
C9	0.0196 (13)	0.0385 (14)	0.0199 (11)	0.0036 (10)	0.0012 (9)	−0.0043 (10)
C10	0.0291 (14)	0.0307 (13)	0.0266 (12)	−0.0061 (11)	0.0073 (10)	0.0002 (10)
C11	0.0222 (12)	0.0257 (12)	0.0157 (10)	0.0002 (10)	0.0037 (9)	−0.0054 (9)
C12	0.0270 (13)	0.0359 (15)	0.0317 (12)	−0.0110 (12)	0.0068 (10)	−0.0065 (12)
C13	0.0214 (12)	0.0316 (13)	0.0204 (10)	−0.0016 (10)	−0.0009 (9)	0.0004 (9)
C14	0.0267 (14)	0.0426 (16)	0.0269 (12)	−0.0030 (12)	0.0021 (10)	−0.0024 (11)
C15	0.0376 (16)	0.056 (2)	0.0256 (12)	0.0101 (15)	0.0042 (11)	−0.0030 (13)
C16	0.0589 (19)	0.0398 (17)	0.0267 (13)	0.0142 (15)	−0.0054 (13)	−0.0064 (12)
C17	0.0522 (17)	0.0255 (13)	0.0324 (13)	−0.0012 (13)	−0.0088 (12)	0.0023 (11)
C18	0.0325 (14)	0.0309 (14)	0.0231 (11)	−0.0050 (11)	−0.0020 (10)	0.0032 (10)

### Geometric parameters (Å, °)

**Table:**

N1—C1	1.320 (2)	C7—H7B	0.9800
N1—C4	1.363 (3)	C7—H7C	0.9800
N2—C2	1.301 (3)	C8—C10	1.528 (3)
N2—C1	1.355 (3)	C8—C9	1.531 (3)
N3—C11	1.357 (3)	C9—H9A	0.9800
N3—C8	1.478 (3)	C9—H9B	0.9800
N3—H3	0.92 (3)	C9—H9C	0.9800
O1—C2	1.341 (2)	C10—H10A	0.9800
O1—C5	1.451 (2)	C10—H10B	0.9800
O2—C3	1.346 (3)	C10—H10C	0.9800
O2—H2	0.87 (3)	C12—C13	1.511 (3)
O3—C6	1.221 (2)	C12—H12A	0.9900
O4—C6	1.320 (3)	C12—H12B	0.9900
O4—C7	1.460 (2)	C13—C14	1.384 (3)
O5—C11	1.211 (3)	C13—C18	1.392 (4)
O6—C11	1.358 (3)	C14—C15	1.391 (4)
O6—C12	1.438 (3)	C14—H14	0.9500
C1—C8	1.530 (3)	C15—C16	1.386 (5)
C2—C3	1.422 (3)	C15—H15	0.9500
C3—C4	1.375 (3)	C16—C17	1.375 (4)
C4—C6	1.490 (3)	C16—H16	0.9500
C5—H5A	0.9800	C17—C18	1.388 (4)
C5—H5B	0.9800	C17—H17	0.9500
C5—H5C	0.9800	C18—H18	0.9500
C7—H7A	0.9800		
			
C1—N1—C4	116.32 (18)	C1—C8—C9	112.28 (18)
C2—N2—C1	117.25 (17)	C8—C9—H9A	109.5
C11—N3—C8	120.17 (17)	C8—C9—H9B	109.5
C11—N3—H3	117.4 (18)	H9A—C9—H9B	109.5
C8—N3—H3	113.6 (19)	C8—C9—H9C	109.5
C2—O1—C5	115.85 (16)	H9A—C9—H9C	109.5
C3—O2—H2	112 (2)	H9B—C9—H9C	109.5
C6—O4—C7	116.03 (16)	C8—C10—H10A	109.5
C11—O6—C12	116.08 (19)	C8—C10—H10B	109.5
N1—C1—N2	125.66 (19)	H10A—C10—H10B	109.5
N1—C1—C8	118.88 (18)	C8—C10—H10C	109.5
N2—C1—C8	115.42 (17)	H10A—C10—H10C	109.5
N2—C2—O1	120.61 (17)	H10B—C10—H10C	109.5
N2—C2—C3	122.69 (19)	O5—C11—N3	126.3 (2)
O1—C2—C3	116.69 (18)	O5—C11—O6	124.3 (2)
O2—C3—C4	127.57 (18)	N3—C11—O6	109.39 (18)
O2—C3—C2	117.10 (18)	O6—C12—C13	112.31 (19)
C4—C3—C2	115.33 (18)	O6—C12—H12A	109.1
N1—C4—C3	122.66 (17)	C13—C12—H12A	109.1
N1—C4—C6	118.47 (18)	O6—C12—H12B	109.1
C3—C4—C6	118.86 (18)	C13—C12—H12B	109.1
O1—C5—H5A	109.5	H12A—C12—H12B	107.9
O1—C5—H5B	109.5	C14—C13—C18	118.8 (2)
H5A—C5—H5B	109.5	C14—C13—C12	122.4 (2)
O1—C5—H5C	109.5	C18—C13—C12	118.8 (2)
H5A—C5—H5C	109.5	C13—C14—C15	120.2 (3)
H5B—C5—H5C	109.5	C13—C14—H14	119.9
O3—C6—O4	124.10 (18)	C15—C14—H14	119.9
O3—C6—C4	121.35 (19)	C16—C15—C14	120.4 (3)
O4—C6—C4	114.55 (17)	C16—C15—H15	119.8
O4—C7—H7A	109.5	C14—C15—H15	119.8
O4—C7—H7B	109.5	C17—C16—C15	119.7 (3)
H7A—C7—H7B	109.5	C17—C16—H16	120.1
O4—C7—H7C	109.5	C15—C16—H16	120.1
H7A—C7—H7C	109.5	C16—C17—C18	119.9 (3)
H7B—C7—H7C	109.5	C16—C17—H17	120.0
N3—C8—C10	106.74 (17)	C18—C17—H17	120.0
N3—C8—C1	109.65 (18)	C17—C18—C13	120.9 (3)
C10—C8—C1	108.25 (18)	C17—C18—H18	119.5
N3—C8—C9	109.79 (19)	C13—C18—H18	119.5
C10—C8—C9	110.0 (2)		
			
C4—N1—C1—N2	2.0 (3)	C11—N3—C8—C10	−168.3 (2)
C4—N1—C1—C8	179.8 (2)	C11—N3—C8—C1	−51.3 (3)
C2—N2—C1—N1	−2.9 (3)	C11—N3—C8—C9	72.5 (3)
C2—N2—C1—C8	179.2 (2)	N1—C1—C8—N3	158.04 (19)
C1—N2—C2—O1	−178.2 (2)	N2—C1—C8—N3	−23.9 (3)
C1—N2—C2—C3	0.9 (3)	N1—C1—C8—C10	−85.9 (2)
C5—O1—C2—N2	2.5 (3)	N2—C1—C8—C10	92.1 (2)
C5—O1—C2—C3	−176.63 (19)	N1—C1—C8—C9	35.7 (3)
N2—C2—C3—O2	−177.9 (2)	N2—C1—C8—C9	−146.3 (2)
O1—C2—C3—O2	1.2 (3)	C8—N3—C11—O5	−19.5 (3)
N2—C2—C3—C4	1.7 (3)	C8—N3—C11—O6	162.23 (18)
O1—C2—C3—C4	−179.13 (19)	C12—O6—C11—O5	−5.0 (3)
C1—N1—C4—C3	1.0 (3)	C12—O6—C11—N3	173.27 (18)
C1—N1—C4—C6	−179.9 (2)	C11—O6—C12—C13	−92.1 (2)
O2—C3—C4—N1	176.9 (2)	O6—C12—C13—C14	9.1 (3)
C2—C3—C4—N1	−2.7 (3)	O6—C12—C13—C18	−169.70 (19)
O2—C3—C4—C6	−2.2 (3)	C18—C13—C14—C15	0.1 (3)
C2—C3—C4—C6	178.2 (2)	C12—C13—C14—C15	−178.7 (2)
C7—O4—C6—O3	−2.9 (3)	C13—C14—C15—C16	−0.2 (4)
C7—O4—C6—C4	176.54 (19)	C14—C15—C16—C17	0.6 (4)
N1—C4—C6—O3	177.4 (2)	C15—C16—C17—C18	−0.8 (4)
C3—C4—C6—O3	−3.5 (3)	C16—C17—C18—C13	0.7 (4)
N1—C4—C6—O4	−2.1 (3)	C14—C13—C18—C17	−0.4 (3)
C3—C4—C6—O4	177.1 (2)	C12—C13—C18—C17	178.5 (2)

### Hydrogen-bond geometry (Å, °)

**Table:**

*D*—H···*A*	*D*—H	H···*A*	*D*···*A*	*D*—H···*A*
O2—H2···O5^i^	0.87 (3)	2.27 (3)	2.889 (2)	128 (2)
O2—H2···O3	0.87 (3)	1.95 (3)	2.652 (2)	136 (3)

Symmetry codes: (i) −*x*+1, *y*+1/2, −*z*+2.
